# Advances in Drug Design and Development for Human Therapeutics Using Artificial Intelligence-II

**DOI:** 10.3390/biom13121735

**Published:** 2023-12-02

**Authors:** Dongqing Wei, Gilles H. Peslherbe, Gurudeeban Selvaraj, Yanjing Wang

**Affiliations:** 1 State Key Laboratory of Microbial Metabolism, Joint International Research Laboratory of Metabolic & Developmental Sciences, School of Life Sciences and Biotechnology, Shanghai Jiao Tong University, Minhang, Shanghai 200240, China; dqwei@sjtu.edu.cn (D.W.); wangyanjing@sjtu.edu.cn (Y.W.); 2 Centre for Research in Molecular Modeling (CERMM), Department of Chemistry and Biochemistry, Concordia University, Montreal, QC H4B 1R6, Canada; gilles.peslherbe@concordia.ca; 3 Bioinformatics Unit, Department of Biomaterials, Saveetha Dental College and Hospital, Saveetha Institute of Medical and Technical Sciences (SIMATS) University, Chennai 600077, TN, India

Building on our 2021–2022 Special Issue, “Advances in Drug Design and Development for Human Therapeutics Using Artificial Intelligence [1]”, our second research collection, which covers the period from 2022 to 2023, delves deeper into this topic by reporting novel studies based on cutting-edge deep learning, neural network, and pharmaco-informatics technologies. In this second Special Issue, we address a broad spectrum of crucial topics, encompassing the prediction of cell-penetrating peptides; the intricate design of proteins; the complex interplay of pathological factors that influence neurorehabilitation; the prediction of drug-likeness; as well as the identification of inhibitors targeting Cdc42-associated kinase (ACK1), human ribonucleotide reductase M1 (hRRM1), and antiviral compounds designed to combat the ever-evolving challenges posed by the SARS-CoV-2 virus. This expanded body of research reflects our unwavering commitment to advancing the the field of drug design and development, with particular emphasis on leveraging the capabilities of artificial intelligence to revolutionize the way we approach human therapeutics.

In the context of drug delivery, it is crucial for therapeutic drug molecules to reach and interact with intracellular organelles like the cytoplasm, nucleus, lysosomes, and mitochondria to achieve their intended therapeutic effects. This necessitates the crossing of the cell membrane. However, hydrophilic therapeutics or large biomolecules, such as proteins or nucleic acids, often struggle to penetrate cell membranes. To address this issue, cell-penetrating peptides (CPPs) come into play. CPPs are typically composed of 4–40 amino acids and possess the unique ability to facilitate the entry of various molecular cargoes into cells. Nevertheless, the identification of specific regions within a protein or peptide sequence with CPP properties is traditionally a laborious and time-consuming process involving experimental verification and optimization. To expedite this process and make it more efficient, Park et al. [2] have introduced a CPP prediction model which leverages deep learning-based natural language processing techniques, shedding light on the essential sequence patterns required to enhance the CPP characteristics. Ultimately, this breakthrough not only streamlines the design of novel CPPs but also enhances their cell-penetrating properties, offering promising enhancements in drug delivery strategies.

Protein sequence design is the intricate process of crafting a sequence of amino acids that, upon folding, assumes a specific shape to carry out a designated biological function. This multifaceted endeavor primarily involves two types of techniques: traditional-physics-based (TPB) and machine learning-based (MLB) methods. TPB methods, although instrumental in laying the groundwork for protein design, have certain limitations. These traditional approaches heavily rely on physical models, contributing significantly to our comprehension of protein structure and function. However, their drawbacks include intricate model complexities, sensitivity to model accuracy, computational resource intensiveness, and often a lack of adaptability. MLB methods, on the other hand, harness the power of machine learning to predict protein structure and function. They differ from TPB methods by not explicitly involving energy calculations. While MLB methods are efficient and capable, they tend to lack interpretability and may have limited explanatory power. This leaves room for uncertainty regarding whether the resulting structure-sequence model effectively optimizes the intended function. To bridge this gap, Omer and coworkers [3] have introduced a hybrid approach, which combines physics-based and deep learning-based techniques. These methods draw from empirical energy functions and machine learning models, respectively. Notably, both approaches are parameterized using known protein structures, regardless of the specific folding and operational conditions. The authors have further enriched their methodology by incorporating molecular dynamics (MD) simulations. These simulations enable the exploration of the protein’s energy landscape, allowing for the design of proteins that approximate the desired structure. Importantly, this approach also facilitates property optimization, even under non-ambient conditions, while accounting for the protein’s inherent flexibility. The fundamental assumption here is that MD simulations accurately describe protein interactions, providing a powerful and adaptable framework for protein design.

Thrombolysis administration typically reduces the risk of death and stroke-related consequences during the acute phase. However, it does not serve as a predictive factor for neurorehabilitation outcomes in the subacute phase, likely due to the complexity of various clinical factors. To address this, Iosa et al. [4] designed an artificial neural network (ANN) method to identify and assign weight to prognostic factors. This study suggests that prognostic factors may differ between patients who received thrombolysis and those who did not. While thrombolysis itself may not independently predict neurorehabilitation outcomes, it appears to influence the relative importance of other clinical factors in predicting the responsiveness of patients to neurorehabilitation.

Activated Cdc42-associated kinase (ACK1) signaling plays a pivotal role in various cellular functions, including growth, proliferation, and migration, involving multiple non-receptor tyrosine kinases. Overexpression of ACK1 has been linked to several cancers, such as prostate, breast, pancreatic, ovarian, lung, gastric, hepatocellular, and renal carcinoma. In the past decade, researchers have developed specific inhibitors for ACK1, which are currently in the pre-clinical stage. Kumar et al. [5] utilized series of pharmaco-informatics techniques to design drug-like ACK1-selective scaffolds for inhibiting ACK1, introducing potential novel therapeutic options.

Computational drug-likeness prediction plays a critical role in evaluating the potential of small molecules to become marketable drugs and is a key metric in screening drug candidates. Initially, these predictions were rule-based, relying on physicochemical properties. Lipinski and coworkers introduced ‘the Rule of 5’ (Ro5) to exclude compounds with poor absorption or permeation, which are unfavorable for drug development. In this study, Cai et al. [6] have introduced a graph neural network-based platform called miDruglikeness, which outperforms quantitative estimate of drug likeness (QED) by quickly assessing a vast amount of molecular data, including those generated by deep generative networks, offering improved predictive accuracy.

Biliary tract cancer encompasses a diverse group of malignant tumors that occur in the biliary tract, ranking as the second most common type of liver cancer. Ribonucleotide reductase serves as a valuable therapeutic target, which is crucial in regulating DNA synthesis and repair. This enzyme uniquely converts nucleotide diphosphates into two deoxyribonucleotide diphosphates. The search for chemical compounds to treat diseases is known for its complexity, challenges, time requirements, and high costs in the pharmaceutical industry. In this study, Islam et al. [7] employed an integrated approach in pharmaco-informatics, utilizing molecular docking, negative image-based ShaEP scoring, absolute binding free energy, in silico pharmacokinetics, and toxicity assessments to design new inhibitors for Ribonucleotide reductase.

In response to the ongoing global challenges posed by the COVID-19 pandemic, Hu et al. [8] have undertaken the development of a multi-task deep learning model. The primary goal of this model is to systematically screen and identify potentially effective inhibitors against SARS-CoV-2, the virus responsible for the disease. The urgency of this endeavor is underscored by the devastating impact of COVID-19, which has resulted in millions of deaths worldwide and continues to infect more individuals due to viral variants and the large population of immunocompromised individuals. To combat this ongoing threat, the authors have used their model to identify promising compounds that have the potential to inhibit the virus. These findings offer a ray of hope and provide valuable insights that can guide subsequent pre-clinical experiments and the development of interventions against the virus.

We anticipate that the diverse range of interdisciplinary subjects explored in this Special Issue will stimulate meaningful dialogue among researchers specializing in artificial intelligence within the realm of drug design and development. Our heartfelt gratitude goes out to all the contributing authors, whose work promises to be an invaluable addition to the field. We would also like to extend our appreciation to the esteemed scientific experts in the fields of artificial intelligence, computational modeling, and drug design for their invaluable comments and suggestions, which have significantly enhanced the overall quality of this Special Issue. Lastly, we wish to express our gratitude to the Editor-in-Chief, assistant editor, academic editors, and the entire editorial team at *Biomolecules* for the opportunity to collaborate, connect with experts in the field and produce an excellent Special Issue.
